# Experiences of young Australian mothers with infant feeding

**DOI:** 10.1186/s12884-022-04796-8

**Published:** 2022-06-15

**Authors:** Christa Buckland, Debra Hector, Gregory S. Kolt, Jack Thepsourinthone, Amit Arora

**Affiliations:** 1 School of Health Sciences, Western Sydney University, Locked Bag 1797, Penrith, NSW 2751 Australia; 2 Health Equity Laboratory, Campbelltown, NSW 2560 Australia; 3 Cancer Australia, Surry Hills, NSW 2010 Australia; 4 Translational Health Research Institute, Western Sydney University, Locked Bag 1797, Penrith, NSW 2751 Australia; 5 Discipline of Child and Adolescent Health, Sydney Medical School, Faculty of Medicine and Health, The University of Sydney, Westmead, NSW 2145 Australia; 6 Oral Health Services, Sydney Local Health District and Sydney Dental Hospital, NSW Health, Surry Hills, NSW 2010 Australia

**Keywords:** Young mothers, Breastfeeding, Infant feeding, Qualitative, Public health, Australia

## Abstract

**Background:**

Despite the overwhelming evidence of health and other benefits of breastfeeding and recommendations to breastfeed by peak health bodies, breastfeeding rates are significantly lower than recommended in Australia and globally. Young mothers are identified as being at high risk of not breastfeeding their infants according to infant feeding recommendations. This study aimed to gain an in-depth understanding of young Australian mothers’ experiences of infant feeding, and to explore the factors which facilitated or hindered adherence to recommended breastfeeding practices.

**Methods:**

Purposive and snowball sampling were used to recruit young mothers in Australia (*n* = 17) aged 18–25 years who had given birth to their first child within the last two years. Data were collected through semi-structured interviews via online videoconferencing. Interviews were audio-recorded, transcribed verbatim, coded, and subsequently analysed. Thematic analysis was conducted using *Quirkos*, a qualitative data management and analysis software.

**Results:**

Four major themes were identified: Breastfeeding is challenging; Feeling judged about infant feeding choices; Experiences with breastfeeding support; and Experiences and views on breastfeeding education. Most mothers reported experiencing breastfeeding challenges, particularly those arising from lactation difficulties, yet many were able to overcome these challenges through their determination to breastfeed. Many mothers expressed perceiving judgement from others for breastfeeding in public. Some mothers who were bottle feeding their infants, whether with expressed breast milk or infant formula, felt that they were being judged for using a bottle. Many mothers reported professional, partner, family, and peer support as critical facilitators to the continuation of breastfeeding. Most mothers shared positive experiences of attending breastfeeding education sessions, but indicated the need for community education to reduce judgement toward mothers’ infant feeding decisions.

**Conclusions:**

The barriers and enablers to infant feeding experienced by young mothers are similar to those experienced by mothers of all ages. Young mothers experience significant breastfeeding challenges and need support from partner, family, peers, and professionals to continue breastfeeding in line with recommendations. Breastfeeding campaigns to support young mothers should be aimed at the wider community to increase awareness of breastfeeding challenges, reduce stigma associated with breastfeeding in public, and to support mothers in their infant feeding decisions.

## Background

Breastfeeding provides short- and long-term health benefits for both infants and mothers, as well as economic and ecological benefits [1, 2]. Breastfeeding has been recognised as an unequalled way of providing ideal food for the optimal growth and development of infants [3]. Breast milk contains numerous biologically active substances and immune-related components, thereby supporting the child’s immune system and providing protection against diseases [1, 2]. Breastfeeding reduces the risk of respiratory and gastrointestinal infections, otitis media, and urinary tract infections [1, 4], improves cognitive development [5], and protects against chronic illnesses such as asthma, obesity, and diabetes [1, 6, 7]. Breastfeeding has been shown to reduce infant mortality rates in developed and developing countries [8, 9]. In addition, research suggests that the influence of breastfeeding on a child’s health and development extend beyond the first and second years of life [1, 10]. Health benefits of breastfeeding to the mother include reduced risk of type 2 diabetes, breast cancer, and ovarian cancer [1, 8, 11], reduced postpartum blood loss, prevention of postpartum depression, and increased time between pregnancies assisting in child spacing [1, 6]. Prior studies conducted in several high-income countries suggest that breastfeeding can help save millions of dollars annually in health care costs [12–14].

The World Health Organization (WHO) recommends that infants be exclusively breastfed until six months of age, followed by introduction of nutritionally adequate and safe complementary foods while continuing to breastfeed for up to two years or beyond [3]. In Australia, the National Health and Medical Research Council (NHMRC) recommends that infants be exclusively breastfed until ‘around’ six months of age when solid foods are introduced, and then for breastfeeding to continue until 12 months of age and beyond, for as long as the mother and child desire [15]. Exclusive breastfeeding, the practice in which an infant only receives breast milk and no other solids or liquids [3], ensures that infants receive the optimal nutritional and additional health benefits of breast milk [15]. Although any amount of breastfeeding is beneficial to the infant and mother, infants who are exclusively breastfed for six months have greater health benefits compared to those who are partially breastfed and/or exclusively breastfeed for three to four months [7, 11].

Despite recommendations to breastfeed due to its multiple benefits, breastfeeding rates are significantly lower than recommended in many parts of the world [16]. Globally only 41% of infants are exclusively breastfed for the recommended six months, over two-thirds of mothers continue breastfeeding for 12 months, and this decreases to 45% by two years of age [16]. In Australia, although most mothers initiate breastfeeding, the breastfeeding rates are far lower than the global average. The 2010 Australian National Infant Feeding Survey (NIFS) [17] indicated that around 60% of infants were still receiving some breast milk at six months, however only 15% of Australian mothers exclusively breastfed for five complete months and only 42% of infants were receiving any breast milk at 7–12 months of age [17].

Young mothers have been identified as being at a high risk of not breastfeeding their infants compared with older mothers in national and international studies [17–19]. According to the 2010 Australian NIFS [17], the proportion of Australian mothers aged 24 years or younger who exclusively breastfed for five complete months was one-third that of mothers aged 35 years or older (6% compared to 19%). Lowest rates of any breastfeeding were observed across all child ages among the youngest mothers, while higher breastfeeding rates were found among older mothers. Only 19% of mothers aged 24 years or younger were breastfeeding their infant at 7–12 months of age compared with 45% of mothers aged 35 years or older [17].

There are few published studies internationally, and even fewer in Australia, that have focused on exploring younger mothers’ experiences of breastfeeding. A recent systematic review identified 22 studies on breastfeeding among teenage mothers and concluded that the key challenges included lack of support, stigma, and inhospitable spaces [20]. Reports on breastfeeding experiences among young Australian mothers are limited [21, 22], with the key issue identified as the stigma of being judged as a young mother. Increased understanding of the experiences of young mothers with breastfeeding will provide insights into the factors that influence their infant feeding decisions, which may in turn guide the development of interventions to support and enable them to breastfeed in line with recommended practices. Therefore, this qualitative study aimed to gain an in-depth understanding of young Australian mothers’ experiences of infant feeding, and to explore the factors which facilitated or hindered adherence to recommended breastfeeding practices.

The following research questions guided this study:

- What are the experiences of infant feeding among young mothers in Australia?
- What are the perceived enablers and barriers to following recommended breastfeeding practices among young mothers in Australia?

## Methods

### Study design

This study used a qualitative research design [23] which allowed for in-depth data collection on the participants’ perspectives and experiences, as well as fostering simultaneous data collection and analysis. Data were collected through semi-structured interviews [24] and conducted between September 2019 and January 2020. The intention was to gain a comprehensive understanding of young mothers’ experiences of infant feeding and to explore the factors which facilitated or hindered adherence to recommended breastfeeding practices.

### Setting and sample

Young Australian mothers aged between 18–25 years who had given birth within the last two years to their first child, were eligible to participate. A purposive sampling technique was used to gain information-rich data [24]. Participants were recruited primarily through posts on Facebook and Instagram. These social media platforms were chosen because of the high number of young female users [25]. Social media is also an effective method for recruiting participants due to the ability to target hard-to-reach populations, and it allows easy sharing of recruitment information among interest and community groups [26]. As the lead researcher has a preestablished network with doulas, midwives, allied health professionals and mothers, the flyer was posted on her personal profile and page in Facebook and Instagram. Social media users shared the post on their pages and profiles, as well as via relevant online groups. The Facebook posts were shared over 80 times and viewed by over 9000 social media accounts. Additionally, relevant community and non-government organisations (including child-care centres) in South Western Sydney, New South Wales, Australia were contacted and sent a flyer and links to the social media posts. Flyers were also physically placed on noticeboards of local community health offices and local child-care centres. Snowball sampling [27] was implemented to enhance the purposive sampling by inviting respondents to refer friends or family who met the inclusion criteria of the study.

A maximum variation sampling strategy was adopted to enrich the data quality, to support capture of diverse dimensions of interest and identification of key patterns in the data [23, 24]. As the intent was to recruit young mothers with a variety of infant feeding experiences, the term ‘infant feeding’ was used in the flyer and social media posts, rather than ‘breastfeeding’. Further, no restrictions were placed on the participants’ region of residence within Australia or their socio demographic characteristics, except for the age of mother (18–25 years) and the age of the child (up to 2 years), to support inclusion of a diverse group of mothers with a broad range of experiences with infant feeding.

All respondents interested in participating in the study contacted the lead researcher via email or telephone call. To ensure confidentiality of all participants, no discussions were held in the social media platforms. The primary researcher (CB) explained the study to each participant and provided them with a participant information sheet and consent form by email. Written informed consent was obtained from each participant, also via email, prior to study commencement. Data collection and analysis were conducted simultaneously, and recruitment of participants continued until it was determined that data saturation had been reached, defined as the point where the addition of new participants did not aid in further collection of new information [23]. An AUD$25 gift voucher, as was indicated on the recruitment flyer, was provided to each participant following the interview to thank them for their time.

### Data collection

In-depth, semi-structured interviews were conducted with each participant by the primary researcher (CB). Mothers could opt to attend in person at a facility at Western Sydney University or participate via online videoconferencing (Zoom). Each interview commenced with open-ended questions on infant feeding to initiate a conversation, put the participant at ease, as well as build rapport. Table 1 shows the semi-structured interview guide used to ensure all topics were covered in each interview. Mothers were able to attend to their babies, when necessary, throughout the interview. The mean length of interview was 57 min. All interviews were audio-recorded and subsequently transcribed *verbatim*. A professional transcription service was employed to ensure accuracy of the verbatim transcriptions of the audio-recordings.

**Table 1:** Semi-structured interview guide

Interview Questions
**Prior to birth**
1. What did you think were the options for feeding your baby, prior to giving birth? What did you know and understand about each option? How did you decide which option was right for you and your baby? Who helped you in making that decision? 2. How did you feel about breastfeeding prior to giving birth? Why did you feel this way? 3. Did you know anyone among your family or friends who has breastfed before?
**After the birth**
1. After the birth, how and what did you feel about feeding your infant (in the hospital, at home, in the community)? How did these experiences affect your decisions around feeding your baby? 2. What challenges did you experience with breastfeeding? Were you able to overcome these difficulties or did they stop you breastfeeding your child? If so, how? 3. What positive factors do you think helped you to breastfeed? 4. Were your family and friends supportive of breastfeeding? How did they support (or not support) you? 5. When did you give your child infant formula or other liquids (such as water or juice)? Was this in addition to breastmilk? Why did you give your child infant formula or other liquids at that time? 6. When did you give your child semi-solid and solid foods? Was that in addition to breastmilk? Why did you give your child solid foods at that time?
**Now**
1. How do you feel about breastfeeding now? Why? 2. Are you aware of the term exclusive breastfeeding? 3. What do you think are the benefits to mothers and to children from exclusive breastfeeding or any breastfeeding? How long do you think mothers should breastfeed for? 4. What do you believe could help young mothers to exclusively breastfeed? 5. Do you have any other thoughts or experiences you would like to share?

### Data analysis

Transcripts were uploaded to the qualitative data management software *Quirkos* (Quirkos, Edinburgh, Scotland, UK), read and reread to gain familiarity with the data, and analysed thematically using an inductive approach to identify and analyse contextual patterns and themes within the data [28]. Three researchers (CB, AA, JT) coded the transcripts and identified themes and sub-themes from the data. Further identification of themes and sub-themes occurred through discussion with all researchers. Direct quotes were identified to support the key themes and sub-themes and are presented in the results.

### Ethical considerations

This study was approved by the Western Sydney University Human Research Ethics Committee (Approval Number H13364). Participants were reminded both verbally and in the written participant information sheet that they could withdraw at any time. The interviews were conducted in a sensitive and professional manner, followed the semi-structured interview guide and were non-judgemental about the infant feeding decisions. Audio-recordings were stored securely in a password protected folder on the primary researcher’s computer. Participants were deidentified in the transcriptions and assigned a numerical key.

## Results

A sample of 17 mothers aged 18 to 25 years at the time of their first baby’s birth participated in this study. The age of their babies at the time of the interview ranged from 3 to 18 months. Ten mothers were recruited through Facebook, five through Instagram, and two through participant referral. Of the 17 participants, the majority (*n* = 11) were of Caucasian ethnicity, and most participants (*n* = 16) were born in Australia. Six participants resided in the Sydney metropolitan area, three in other areas of New South Wales (the most populous state in Australia), five in the state of Queensland, and three in the state of Victoria. The mothers had a broad range of education levels and work backgrounds. A summary of the sociodemographic characteristics of the participants is presented in Table 2.

**Table 2:** Sociodemographic characteristics of the study participants (*n* = 17)

Characteristics^a^	*n* (%) or Mean ± SD
Age of mother at first baby’s birth (years)	22.8 ± 1.9
Age of infant at interview (months)	6.2 ± 4.6
Country of birth	
Australia	16 (94.1)
Overseas	1 (5.9)
Ethnicity	
Caucasian	11 (64.7)
Non-Caucasian	6 (35.3)
Education	
Bachelor degree/above	6 (35.3)
Diploma/certificate	5 (29.4)
Year 12 or below	4 (23.5)
Relationship status	
Married/living with a partner	16 (94.1)
Single	1 (5.9)
Resided in	
New South Wales (NSW)	9 (52.9)
Queensland (QLD)	5 (29.4)
Victoria (VIC)	3 (16.6)
Mode of delivery	
Vaginal birth	13 (76.5)
Caesarean birth	4 (23.5)

^a^The total for some categories is not 17 due to missing data

Sixteen mothers participated in the interviews via online video conferencing (Zoom) and one mother participated in a face-to-face interview at Western Sydney University. All mothers attended to their babies, when necessary, throughout the interview. All young mothers in this study had initiated breastfeeding, and the participants included mothers who were continuing to breastfeed as well as those who had ceased breastfeeding. Infant feeding practices included breastfeeding and/or bottle feeding, the latter with either expressed breast milk or formula.

Four major themes were identified from thematic analysis of the data: (1) Breastfeeding is challenging; (2) Feeling judged about infant feeding choices; (3) Experiences with breastfeeding support; and (4) Experiences and views on breastfeeding education. The themes and sub-themes are presented in Table 3.

**Table 3:** Themes and sub-themes

Major Themes	Sub-Themes
Theme 1: Breastfeeding is challenging	Lactation difficulties
	Complexity of breastfeeding aids
	Determination to breastfeed
Theme 2: Feeling judged about infant feeding choices	Breastfeeding in public
	Bottle/formula feeding
Theme 3: Experiences with breastfeeding support	Professional support
	Partner support
	Family support
	Peer support
Theme 4: Experiences and views on breastfeeding education	Prenatal education
	Early postnatal education
	Community education

### Theme 1: breastfeeding is challenging

All the young mothers described facing various challenges when attempting to breastfeed. Some mothers were able to overcome these challenges and described a positive relationship with breastfeeding at the time of the interview. Others were not able to overcome their challenges and had ceased breastfeeding. The mothers who had ceased breastfeeding shared varying feelings about their experience. Some mothers were content with what they were able to achieve despite having ceased breastfeeding, whereas others were dissatisfied with their experience. Three sub-themes were identified: Lactation difficulties; Complexity of breastfeeding aids; and Determination to breastfeed.

#### Lactation difficulties

Several difficulties related to lactation were described by the participants. These included challenges relating to latch, pain, and supply – often the challenges were interrelated. For example, nipple pain as a result of latch issues impacted the supply of breast milk. One participant attributed cessation of exclusive breastfeeding to lactation difficulties, and indicated that hospitalisations due to mastitis was the primary reason for stopping breastfeeding.

*I think I breastfed for five months, but she wasn’t exclusively breastfed, in that we came home on formula, because I couldn’t produce enough milk. I couldn’t get her to latch, like it was just not happening. So then she was formula fed, breastfed, and I pumped. That was for five months. But then at about three months in, I got severe mastitis, and ended up in hospital for, I think three times for it. Then by five months I just went, “I can’t do this anymore.” I actually stopped breastfeeding, and then she was solely formula fed from then on out (P-14, 24 years old).*

#### Complexity of breastfeeding aids

The participants described various experiences they had with breastfeeding aids (e.g., nipple shields, breast pumps). For some mothers, nipple shields provided a solution to the latch and pain difficulties. These mothers expressed frustration that the nipple shields were not offered sooner by health professionals.

*Well, I begged for a nipple shield. And I got one at the end of that class. I think after a couple of rounds of tears, they were like, “Oh, she needs a nipple shield.” I’m like, “Yeah.” (P-13, 24 years old).*

Other mothers, however, felt that nipple shields complicated breastfeeding and made it less satisfying. Some mothers shared their experiences of how the constant application and then the removal of the shield by their baby interrupted breastfeeding.

*At first, it was very tricky … I found it really annoying that every time I put it on, when I get my baby to suck on it, he pulls it off. Like he, and it comes off. Then the milk comes out, it will just fall out everywhere. Oh my God! Put the baby down, put it back on, put him back on the boob again. And then he drops it, and it’s a back-and-forth situation (P-15, 20 years old).*

Some participants expressed that breast pumps were helpful and having a good quality pump, although expensive, is important. One participant pointed out that some younger parents may not be able to afford a good quality pump.

*They’re quite pricey, I think they were over $200 for that. But it does make a difference, having a good quality pump, compared to just a cheap version. I think that is important, and I also think that a lot of younger parents aren’t financially able to go and spend that money on something like that, which is, I don’t know, something that’s important, I think (P-16, 20 years old).*

#### Determination to breastfeed

Most mothers experienced some difficulties with breastfeeding. Despite these challenges, many participants described their determination to continue breastfeeding. These mothers credited their breastfeeding success to their determination to breastfeed. Determination was often described as an inner resource which the participant identified as part of their personality.

*I suppose just the fact that I’m really determined to do what I want to do as a person. I was like I’m going to do this and I’m going to get through it (P-10, 22 years old).*

Awareness of the benefits of breastfeeding was a contributing factor to the mothers’ determination to breastfeed. Mothers valued the health benefits of breastfeeding, such as nutrition, immune boosting, antibodies, and colostrum. Some mothers also described breastfeeding as less expensive and more convenient.

*Yeah, that was a big deciding factor.…I was aware of some of the more interesting aspects of it. Like, the way your body can adjust your supply to how much your baby’s asking for it. And if it’s hotter then your baby will drink more and get more watery milk than fatty milk to keep them hydrated. Things like passing antibodies and helping their immune systems. I supposed I was being health inclined, interested in that side of things. And so, was aware of those benefits, which helps make choosing that a bit easier (P-03, 24 years old).*

### Theme 2: feeling judged about infant feeding choices

Feeling judged about their infant feeding choices was a common perception among the participants, regardless of whether they were currently or previously breastfeeding or formula-feeding their infants. Two sub-themes were identified: Breastfeeding in public; and Bottle/formula feeding.

#### Breastfeeding in public

Some mothers described feeling stared at and receiving unwanted comments from strangers when breastfeeding in public. They felt that they were being judged about the appropriateness of the infant’s age for breastfeeding and expectations to “cover up”. Some mothers navigated these interactions confidently while others found feeding in public to be stressful. Some mothers tried to comply with the perceived social expectations to breastfeed discreetly.

*I always thought, “Oh, I’ll just feed him.” But then when everyone’s around you’re a bit like, “Well, I don’t really just want to feed him.” Not even that I’m uncomfortable. Like you notice that everyone else feels a little bit uncomfortable with you doing it. I was like, “I’ll just try and do it discreetly as I can” (P-10, 22 years old).*

The young mothers in this study expressed perceiving judgement from peers, family, and community members. Older community members were perceived to be the main source of judgement.

*I have found that most of the judgment has come from the older generation, when it comes to me going out in public. They are very nice, but you can also sense that if you do something that’s not to their standard, they will judge you. Most other mums, like my age mums who have kids, most are willing to just help you if you need it (P-14, 24 years old).*

One mother expressed concern about breastfeeding in public or when they were around their friends who did not have babies. The participant also suggested that the reason for other people feeling uncomfortable with breastfeeding may arise from ignorance of the biological role of breasts as a food-source for infants, as opposed to a sexual role.

*Reduce the stigma around it. I think a lot of young women in particular feel uncomfortable breastfeeding in public. And a lot of my friends don’t have babies, so their responses to breastfeeding can make you feel uncomfortable breastfeeding in public and/or around them. So just, yeah. Reducing this thing of like boobs are a sexual thing, not baby’s food (P-02, 23 years old).*

#### Bottle/formula feeding

Some mothers felt that people constantly made judgements on those who formula feed their baby. One participant suggested that other people may think that mothers often choose formula feeding as an easier alternative to breastfeeding. Some mothers who were bottle feeding their infants, whether with expressed breast milk or formula, felt that they were being judged for using a bottle and for “giving up”. One participant felt that as compared to mothers who breastfeed, those who feed expressed breast milk via a bottle also receive similar judgement to those who feed formula via a bottle.

*I think there’s a lot more judgment on formula feeding. I think people just think they want the easy way out, but I don’t think there is an easy way out. I think formula feeding is just as hard. So, I feel like there is a lot of judgment on that or judgment on mums that pump and feed as opposed to just feed. I think that's a very modern way of breastfeeding, which I think is fine, but I think there’s a lot of judgment on that as well (P-12, 24 years old).*

### Theme 3: experiences with breastfeeding support

Breastfeeding support was discussed extensively by the participants. The mothers described various experiences with health professionals, intimate partners, family members, and peers, which they perceived to be important. Four sub-themes were identified: Professional support; Partner support; Family support; and Peer support.

#### Professional support

Professional support, including from midwives, lactation consultants, community nurses, and doulas, was perceived to be important and comforting by most mothers.

*So it’s nice to just chat to someone that you know is a professional because you can talk to your mum and you can talk to your partner and they can offer really great support but knowing that it’s professional support is really like, I don’t know I guess comforting, reassuring so yeah (P-07, 22 years old).*

Mothers shared their positive experiences of the breastfeeding support they received at the hospital through a lactation consultant and at home through visits by a midwife. Overall, in-home support, particularly in the first six weeks, was perceived by most mothers to be more personalised and helpful than hospital support.

*I felt like it wasn’t rushed, she actually took the time to listen to me and have a look and provide support … She would just give me a range of options, like if she’s not feeding, you should try this or you should try that. … So she gave me a lot of tips and tricks which helped a lot, which I hadn’t received at the hospital (P-12, 24 years old).*

#### Partner support

Partner support was perceived as a critical facilitator to the continuation of breastfeeding in the first six weeks, especially if the mother was recovering from a caesarean birth. One participant indicated that caesarean section did not have a significant impact on breastfeeding, however, reported that she would be formula feeding if she had not received the same support and help from her partner during her recovery.

*One thing was I’d had a C-section…I don’t think it really affected feeding too much…I would’ve given up within a week of being home if he wasn’t there. If he’d gone back to work after a week, I would be formula feeding now… I just was on the couch for six weeks straight feeding the baby and he literally did everything else. Did all the meals, cleaned the house, did the laundry, nappy changes, everything (P-13, 24 years old).*

One mother noted that her partner’s attitude towards breastfeeding, involvement in infant feeding choices, and overall support influenced her confidence of breastfeeding in public.

*But I think the biggest thing is that if I’m out and about and he gets hungry, my partner is so supportive. He just goes, “Nope, just feed him. If anyone says anything, I’ll just tell them you’re feeding your baby and stuff.” He’s very supportive of like, “This is just how we’re doing it. If anyone says anything just send them to me.” Which is very helpful (P-10, 22 years old).*

#### Family support

Extended family were also a source of support for the participants in the continuation of breastfeeding, particularly if the family members had a personal experience with breastfeeding. Some mothers indicated that they would often reach out to these family members if they had any breastfeeding-related queries.

*Both my mum and my partner’s mum were always very supportive, and his cousin [name], in just if I ever had questions about how he was feeding and stuff, I could ask all three of them … just having those people who I knew had already breastfed was really useful because any question they’d just answer straight away for me. … And they would never judge me so that was good (P-10, 22 years old).*

#### Peer support

Most mothers indicated that they found their peers to be supportive, including other mothers from support groups, friends who have or have not given birth, and mothers in the community who are of a similar age. Peers who had not given birth were generally supportive but less educated on breastfeeding. Some mothers valued peer support over professional support. These mothers expressed a need to connect with others they could relate to.

*I guess having younger people that you can relate to and speak to is really good. Yeah, personally, I’ve found that relating to younger people who I know that breastfeed has been easier than speaking to a professional, I guess (P-12, 24 years old).*

### Theme 4: experiences and views on breastfeeding education

Breastfeeding education that participants had received differed in terms of timing and duration of classes. Many mothers had positive experiences attending breastfeeding education sessions either before or after the birth. While partners were welcome to the sessions, most were attended solely by the mothers as the classes were not at a convenient time for working partners. Views and experiences of breastfeeding education depended on the types of education; prenatal classes, postnatal classes, and community education. Three sub-themes were identified: Prenatal education; Early postnatal education; and Community education.

#### Prenatal education

Prenatal education classes provided information to mothers about the benefits of breastfeeding, breastfeeding techniques, and troubleshooting. It was reported that this helped mothers to decide if they wanted to breastfeed and improved their confidence in the process. Classes ranged from a brief 30-min information session to a two-hour class.

*During the pregnancy I also went to a breastfeeding class, which the lactation consultant at the hospital ran. And that was a two-hour thing. And that was really, really helpful in sort of making me feel comfortable in sort of making that decision (P-09, 25 years old).*

Some mothers found prenatal classes helpful, however others found the classes had too much information and were overwhelming. They also viewed the classes as inadequate without follow-up support from health professionals.

*I found the class beforehand really helpful. Just in having a bit of an idea of what to expect. But at the same time, you don’t want to bombard people with what can go wrong before you’ve even begun. You want to start with a really positive, you can do this, sort of attitude. But then I think in terms of breastfeeding can be really hard. And so, I definitely think that first off education probably isn’t enough to keep people going. You need to be there to follow up. If or when things do happen (P-03, 24 years old).*

#### Early postnatal education

Postnatal education usually referred to an in-hospital session for mothers who had just given birth, facilitated by a lactation consultant midwife, and may have been held at either a set time or operated as a drop-in centre for peer support. Mothers often found postnatal education more practical, allowing them to ask questions directly related to their circumstances and experiences with their baby. Some mothers also felt that taking a class after the birth would be more beneficial than before birth, as it would be easier to learn and figure things out together with the baby.

*To be honest, I’d say after birth because as much as you learn about it before birth, you've got no idea and they just ... They don’t really teach you. They’ll show you, “Oh, this is how a baby’s meant to latch”, and you look at that and then when they’re born, you’re just like, “Oh, okay, well I’ve seen that in my class, this is how it’s meant to latch.” But you really have no idea. I feel like it would be more beneficial after birth to take a class, maybe in the first day that you’re in the hospital or something. I think that would be a lot better, once you’ve got your baby and you’re trying to figure it out together, whereas before you just have no idea, it’s just yourself (P-12, 24 years old).*

One mother felt completely overwhelmed after the birth and was unable to attend the breastfeeding sessions. Another mother reflected that education sessions should include information on all feeding options rather than focusing solely on breastfeeding, such that they would feel comfortable picking another option, if required, in the future.

*So if they turned around and said something about bottle feeding, you know, “If this doesn’t really work for you then it’s okay for you to pick another option.” It would have made me feel a little bit more content. Even at that point, I wasn’t even considering but knowing that at the back of your mind. It would have made me feel a little bit better about making that decision in the future if I needed to (P-05, 24 years old).*

#### Community education

Participants expressed the view that it would be helpful if the community, in general, were better educated on issues related to infant feeding. Suggestions for educating the community included education in schools or through online campaigns.

*I think it’s coming from both. I think that young women that don’t have children, it feels uncomfortable or feel as well that breastfeeding is gross possibly, but definitely education in schools or just online of that it’s okay and that it is the best option and that support to do so. Yeah. Yeah. Definitely just education (P-02, 23 years old).*

Some mothers expressed the need for education to focus on various feeding options depending on the family circumstances to reduce judgement in the community toward mothers’ infant feeding decisions.

*I think just educating and like I said, on the modern ways of feeding a baby and how each one could work for different people based on circumstances, situations. These day and ages, a lot of mums do have to return to work unfortunately and they’re not able to be with their baby 24/7, therefore restricting their feeding options. So, I think if people are more educated on there’s not an ideal mum now, whereas before it was just like a stay at home mum was a thing. Now everything’s changed, so I think feeding’s changed so people need to be aware of that (P-12, 24 years old).*

## Discussion

The findings of this study provide valuable insight into experiences of infant feeding, and breastfeeding in particular, among young mothers in Australia. Various factors which facilitate or hinder adherence to recommended breastfeeding practices were identified. Most mothers reported experiencing several breastfeeding difficulties, with some being able to overcome these challenges through their determination to breastfeed. Most mothers perceived judgement from others for breastfeeding in public or for bottle feeding their infants. Many mothers perceived professional, partner, family, and peer support as critical facilitators to the continuation of breastfeeding. These mothers also shared positive experiences of attending breastfeeding education sessions although indicated the need for community education to reduce judgement toward mothers’ infant feeding decisions.

Most young mothers in this study reported experiencing lactation difficulties such as latching problems, nipple pain, and low milk supply, which were also often interrelated. Research indicates that some of these lactation difficulties may be rectified through education on correct latch and infant positioning [29, 30]. While breastfeeding aids such as breast pumps and nipple shields were reported to be helpful, and solutions to latch and pain difficulties, some mothers indicated that pumps and shields added further complications and barriers to breastfeeding. Common complications associated with nipple shields include inconvenience, falling off the breast, and messiness [31]. Additionally, one participant suggested that a good quality breast pump can be expensive and inaccessible for young mothers. A free breast pump service could be an acceptable incentive intervention to improve breastfeeding outcomes, particularly for low-income mothers [32].

Despite the breastfeeding difficulties, many mothers indicated that they were able to overcome these challenges through their determination. This supports findings from several prior studies [33, 34]. Maternal breastfeeding self-efficacy – the combination of sense of strength, determination, and confidence in maternal ability to continue breastfeeding – can be enhanced through social and health professional support, antenatal breastfeeding education sessions, and self-determination enhancement programmes [35, 36]. In addition, several mothers reported the benefits of breastfeeding as a reason for their determination to continue breastfeeding, as noted in other studies [33]. Increased and continued promotion of the short- and long-term health benefits of breastfeeding is essential to strengthen mothers’ determination to continue breastfeeding [33, 36].

There exists a common concern among mothers, of breastfeeding in public [37, 38]. Young mothers in the current study described feeling judged for breastfeeding in public and indicated trying to breastfeed discreetly (as per the perceived societal expectations). A recent Australian study [39] highlighted that social norms require women to be discreet while breastfeeding in public by covering up, feeding in a location deemed appropriate to avoid causing discomfort to others, and guarding themselves against any judgement. The stigma associated with breastfeeding in public can cause mothers to feel isolated and discouraged from breastfeeding [38]. One mother in this study suggested that others may feel uncomfortable with young mothers breastfeeding in public (or around them) due to the ignorance of the biological role of breasts, as opposed to a sexual role. In Western society, breasts are often objectified and sexualised to such an extent that the maternal role is almost excluded and overlooked [37]. Broader public health efforts and campaigns aimed at the general public may address the negative societal attitudes around breastfeeding in public [37].

In this study some young mothers who were bottle feeding their infants, whether with expressed breast milk or formula, felt they were being judged for using a bottle. The WHO’s definitions [3] for exclusive and any breastfeeding specify the receiving of breastmilk by the infant, which includes expressed breastmilk. A review article [40] reported that mothers who feed their infants with expressed breastmilk are not acknowledged nor specifically supported by the customary channels for new mothers such as infant feeding and government health organisations. Young mothers are an at-risk population to cease breastfeeding early and therefore require targeted support strategies to improve their adherence to infant feeding recommendations [40].

Some mothers reported feelings of disappointment when breastfeeding was not able to be maintained, with some feeling that they were judged for ‘giving up’ and using infant formula. The perceived judgement may stem from societal values, public health discourses, cultural norms, and ideals of ‘good motherhood’, which are often reinforced by hospital practices and public health campaigns [41, 42]. While it is essential to support mothers to make informed decisions about infant feeding, it is more important for health professionals to support these mothers in their decisions and reassure them that their value of being a ‘mother’ is not explicitly linked to their feeding method [41, 43].

Support from professionals and peers were indicated by the young mothers in this study to positively influence infant feeding decisions and continuation of breastfeeding. However increased accessibility and availability of professional support is needed to meet mothers’ individual needs and to provide continued breastfeeding support [44]. Most mothers reported a need and preference for in-home support, especially in the first six weeks’ postpartum, correlating with findings from a systematic review of interventions designed to promote exclusive breastfeeding [45]. Previous studies have also indicated that lactation consultants, community nurses, and doulas delivering in-home postnatal support facilitated positive breastfeeding outcomes and may help mothers meet the breastfeeding guidelines [46, 47]. Furthermore, the finding that peers (i.e., friends, mothers in support groups, and mothers in the community) were an essential source of support for many of the mothers in this study resonates with the results of our recent systematic review, which concluded that peer counselling was the most promising intervention to increase exclusive breastfeeding rates among young mothers [48]. Peer counselling interventions (both face-to-face and web-based) targeting young mothers, utilising peers who were unknown to the participants, have demonstrated a positive effect on breastfeeding rates and duration and could be particularly helpful for younger mothers [49, 50].

In this study, partners and family members were identified as critical facilitators to the continuation of breastfeeding. As mothers with partners who are supportive of breastfeeding are more likely to breastfeed for a longer duration [18, 51, 52], it is vital to educate partners and include them in infant feeding decisions and breastfeeding promotion interventions. However, there is a low uptake among Australian men in utilising breastfeeding-supportive policies such as parental leave [53]. Uptake could be improved by normalising parental leave by partners and providing flexible work options to meet their caring requirements [53]. Other family members, especially the infant’s grandmothers, were also identified as a source of support for the continuation of breastfeeding among these young mothers, and as reported in several earlier studies [18, 51]. When grandmothers are supportive of breastfeeding, they are more likely to be encouraging, resulting in longer breastfeeding duration [51, 54]. The education and counselling of significant family members on the benefits of breastfeeding has been suggested as part of breastfeeding promotion and support interventions [55].

The young mothers in this study described their experiences of breastfeeding education, including prenatal and early postnatal education, as generally positive. Studies have shown that breastfeeding classes either in the prenatal or postnatal period have a positive influence on breastfeeding duration [56, 57]. Criticisms raised by the mothers around these classes such as overload of information and inconvenient class times for their working partner, may be resolved through the tailoring of sessions to mothers’ individual needs [58]. Additionally, the young mothers in our study expressed the need to educate the community on various infant feeding options, to reduce the judgement on mothers’ decisions. Breastfeeding education classes need to foster an inclusive learning environment and address all infant feeding options positively, presenting breastfeeding, expressing, and formula options as legitimate choices [41, 59]. Mothers who bottle feed their infants with either breast milk or formula represent a significant proportion, emphasising the need to address infant feeding options in a balanced and women-centred manner [60].

This study explored the infant feeding experiences of young mothers to better understand the unique barriers and facilitators to breastfeeding for this population group. It was expected that there would be unique factors affecting young mothers which may help explain the lower rates of breastfeeding. However the breastfeeding experiences described by the young mothers in our study were similar to those reported in studies among mothers of all ages [33, 34, 37, 38]. The study findings suggest that while mothers of all ages experience a range of breastfeeding issues, some of these issues may be affecting younger mothers to a greater extent than older mothers. A study conducted in the UK [61] reported that attitudes to breastfeeding differed significantly between older and younger mothers, as did self-efficacy scores. Targeting the attitudes and self-efficacy of younger mothers may therefore be beneficial. Targeted support during the antenatal period or even prior to pregnancy, such as education in schools, has been suggested [35, 36]. Additionally, peer support may be especially important for young mothers as they are less likely to have peers in their existing social network who have breastfed, given that the average age of first-time mothers in Australia is 29.3 years old and only 13% of first-time mothers are less than 25 years old [62]. Targeted support and interventions for young mothers may therefore be beneficial.

### Study strengths and limitations

The current study has several strengths. The qualitative approach enabled the collection of information-rich data about young mothers’ experiences and views of breastfeeding [23]. Open-ended questions were used to enable the participants to share what was important to them. As participants were first time mothers, the qualitative data generated from this study were not influenced by previous breastfeeding experiences. Additionally, the sample size (*n* = 17) was adequate to achieve data saturation, as all dimensions of interest were explored [23]. Finally, this is one of the few studies in Australia to explore breastfeeding experiences among young mothers, a hard-to-reach population group in breastfeeding research.

The current study has several limitations. While most young mothers had infants aged less than six months, the two mothers who had older infants may have experienced issues recalling their experience during early infant feeding. Evidence suggests however that maternal recall of breastfeeding experiences is trustworthy even after a prolonged period of three years [63]. Most mothers were Caucasians and therefore the breastfeeding experiences may not be representative of young mothers from ethnic minorities in Australia, as breastfeeding experiences may vary according to culture, region, and demographics [18]. Although an accuracy check by participants would have enhanced the rigour of this study, the interview transcripts were not provided to participants for checking, primarily due to time constraints and delays due to COVID-19. Potential limitations in the categorisation and interpretation of data in thematic analysis were mitigated as far as possible, by the experience of the research team in qualitative research. While mothers in this study expressed several positive experiences with breastfeeding, the effect of breastfeeding on mother-infant relationships were not explored, which could be addressed by future research.

## Conclusions

The experiences on infant feeding shared by the young mothers in this study indicate a wide range of seemingly critical factors that either facilitated or hindered adherence to recommended breastfeeding practices. Young mothers in Australia in this study reported significant challenges and enablers to breastfeeding. However, it is not clear whether these younger mothers experience these challenges and enablers to a different extent than older mothers or whether these challenges impact on them more, as the challenges and enablers to breastfeeding were those commonly reported among mothers of all ages. Determination to breastfeed is a common theme in the literature regarding the continuation of breastfeeding, especially exclusive breastfeeding, indicating that interventions to improve self-efficacy are required. Professional, partner, family, and peer support of breastfeeding was identified by the young mothers as important, and there is a need to educate fathers, grandmothers, and other family members on the benefits of breastfeeding and inclusion in breastfeeding support interventions. Wider public awareness of the benefits yet challenges of breastmilk feeding, especially via the breast, may support more positive social norms of the variety of infant feeding choices. Peer support appears to be a particularly promising avenue of breastfeeding support for younger mothers.

## Data Availability

The data generated and analysed during the current study are not publicly available due to privacy but are available from the corresponding author on reasonable request.
